# Contrast-Induced Encephalopathy After Coronary Angiography With Low-Osmolar Contrast

**DOI:** 10.1016/j.jaccas.2025.106115

**Published:** 2025-11-20

**Authors:** Anastasia Proshkina, Jonathan Shpigelman, Mohamad Bahrou, Abdul Moiz Hafiz, Ahmad Al Turk

**Affiliations:** a Department of Internal Medicine, Southern Illinois University School of Medicine, Springfield, Illinois, USA; b Division of Cardiology, Southern Illinois University School of Medicine, Springfield, Illinois, USA

**Keywords:** contrast media, encephalopathy

## Abstract

Iatrogenic contrast-induced encephalopathy related to the use of iodinated contrast media is a rare neurologic complication. It tends to be self-limiting and has most commonly been reported with high-osmolar agents. However, it may also occur with low-osmolar, nonionic contrast media during coronary angiography, agents that are more commonly used in current clinical practice for medical imaging. We present 3 cases of contrast-induced encephalopathy that occurred temporally very close to each other in a single-center high-volume cardiac catheterization laboratory among a series of 21 consecutive angiograms performed at our institution over a 1-week period.

Contrast-induced encephalopathy (CIE) is a rare but recognized complication after intra-arterial administration of iodinated contrast during angiographic procedures. Clinical presentations vary widely and can include confusion, altered mental status, hemiparesis, aphasia, seizures, and visual disturbances.^1^ Symptoms typically develop minutes to hours after contrast exposure and resolve spontaneously within 24 to 72 hours, though prolonged and permanent deficits have been described.^2^ The pathophysiology of CIE is not fully understood but is thought to involve a transient disruption of the blood-brain barrier (BBB), potentially leading to contrast-induced neurotoxicity, cerebral edema, and cortical irritation or vasospasm.^3^ Contributing risk factors may include contrast hyperosmolarity, larger contrast volume, and comorbidities including hypertension and renal dysfunction.^4^ The incidence of CIE has been estimated at 0.3% to 1.0%, with most cases involving hyperosmolar, ionic contrast agents.^5^ We describe a series of 3 patients who developed CIE after diagnostic coronary angiography or percutaneous coronary intervention (PCI) using the low-osmolar, nonionic contrast agent, iohexol (Omnipaque 350, GE HealthCare). These cases were identified from a convenience sample of an estimated 21 coronary angiograms using iohexol over a 1-week period at our institution.

**Take-Home Messages**

• Contrast-induced encephalopathy, which may present with a broad spectrum of symptoms, is more commonly associated with high-osmolar, ionic contrast agents but can also occur with low-osmolar, nonionic agents such as iohexol—even at relatively low volumes (eg, 35-50 mL), although higher doses may correlate with more severe presentations.
• As contrast-based procedures become increasingly common, clinicians should maintain a high index of suspicion for contrast-induced encephalopathy, particularly in patients with identifiable risk factors, to avoid misdiagnosis and overtreatment.

## Patient 1

A 73-year-old man with hypertension, hyperlipidemia, and asthma presented to the outpatient clinic with complaints of exertional dyspnea. The follow-up exercise nuclear stress test showed diffuse ST-segment depressions (≥1 mm) with ST-segment elevation in aVR during recovery, with normal myocardial perfusion imaging, interpreted as a moderate-risk finding suggestive of multivessel coronary artery disease (CAD) or left main disease, prompting recommendation for further testing. Coronary computed tomography (CT) angiography was then obtained, which revealed severe right coronary artery (RCA) and left circumflex artery stenosis. On the basis of this presentation, he underwent elective coronary angiography, which demonstrated nonobstructive coronary disease: 20% stenosis in the mid–left anterior descending artery (LAD) only, while the RCA was noted to be tortuous with a horizontal takeoff. A total of 35 mL of iohexol contrast was administered during the procedure. The periprocedural estimated glomerular filtration rate (eGFR) was 71 mL/min/1.73 m^2^, and the peak blood pressure (BP) was 168/107 mm Hg. Approximately 30 minutes after the procedure, the patient reported a 30-minute episode of visual disturbance described as colorful halos in both eyes, later predominantly in the left. He noted blue lights that changed shape. He denied any associated headache or prior migraine history. Stroke evaluation was initiated, with a National Institutes of Health Stroke Scale score of 0 and unremarkable results of noncontrast CT head and magnetic resonance angiography (MRA) of the head and neck. Symptoms resolved spontaneously, and the patient was discharged the same day after observation.

## Patient 2

A 61-year-old woman with CAD status after PCI, hypertrophic cardiomyopathy, heart failure with preserved ejection fraction, diabetes mellitus secondary to partial pancreatectomy, hypertension, hyperlipidemia, and obstructive sleep apnea presented with chest pain and was diagnosed with a non–ST-segment elevation myocardial infarction. She underwent PCI of the LAD with administration of 160 mL of iohexol. The periprocedural eGFR was 51 mL/min/1.73 m^2^, and the peak BP was 192/113 mm Hg. Immediately after the procedure, she developed headache, agitation, dysarthria, aphasia, and altered mental status. The results of CT head, magnetic resonance imaging brain, and both CT angiography and MRA of the head and neck were all negative. Neurologic symptoms resolved gradually over 72 hours without intervention. She was then discharged home in a stable condition on the fourth day after her admission.

## Patient 3

A 65-year-old woman with prior stroke, hyperlipidemia, hypertension, and a 30 pack-year smoking history presented for elective coronary angiography after a 2-year history of intermittent burning, substernal chest pain. She underwent coronary CT angiography, which showed a calcified plaque in the proximal LAD, resulting in a 59% area narrowing, as well as a mildly calcified plaque in the proximal RCA. She subsequently underwent invasive coronary angiography—during which 50 mL of iohexol was administered—which confirmed nonobstructive CAD with 40% stenosis in her mid-LAD. The periprocedural eGFR was 82 mL/min/1.73 m^2^, and the peak BP was 157/98 mm Hg. Immediately afterward, the patient experienced “white spots” in both visual fields lasting approximately 15 minutes, followed by a second 20-minute episode accompanied by headache. She remained neurologically intact, with a National Institutes of Health Stroke Scale score of 0. The results of CT head were negative, and MRA brain revealed a punctate focus in the left cerebellar hemisphere, suspicious for artifact or subacute infarct, because the location did not explain the current symptoms. Symptoms resolved completely without recurrence, and she was discharged the following day.

## Discussion

Omnipaque 350 was approved by the Food and Drug Administration in 1985, and beginning in 2022 its labeling was updated to warn of the potential for CIE with intraarterial or intravenous administration.^6^ The exact incidence of this remains unknown, although it has been estimated to be around 0.3% to 1.0%.^4^ CIE is an under-recognized side effect of iodinated contrast exposure during cardiac catheterization. Its presentation ranges from mild symptoms, such as transient visual disturbances—as seen in 2 of our cases—to more severe symptoms, including altered mental status and expressive aphasia, as observed in case 2. A comparative summary of the 3 cases is provided in Table 1. In all cases, symptoms emerged within minutes of contrast administration and had spontaneous resolution. The proposed pathogenesis centers on transient BBB disruption, which may be consequent to downregulated endothelial tight junction protein expression, direct chemotoxicity to cerebral microvasculature, and/or osmotic stress that dehydrates endothelial cells and separates tight junctions.^3^^,^^5^^,^^7^^,^^8^ These changes may permit contrast extravasation into the brain parenchyma, resulting in cerebral edema, cortical irritation, and transient neuronal dysfunction.^3^ High contrast load is an important risk factor in the development of CIE and likely affects symptom severity.^4^^,^^9^ As mentioned previously, case 2 involved the highest dose of contrast (160 mL) and developed the most prolonged and severe neurologic symptoms. Additional patient-specific risk factors, such as hypertension and renal dysfunction, were present in all 3 cases.^4^ Prior stroke history, as in case 3, has also been shown to increase the risk of CIE, possibly due to BBB compromise from prior injury.^9^ Hypertension may impair cerebral autoregulation and weaken the BBB, whereas renal dysfunction delays contrast clearance, increasing exposure duration.^3^^,^^10^ Case 2 also had the highest BP and the worst renal function, which may have contributed to the severity of her symptoms.

**Table 1:** Patient Characteristics and Case Comparison

	Patient 1	Patient 2	Patient 3
Age (y)	73	61	65
Sex	Male	Female	Female
Hypertension	Yes	Yes	Yes
Hyperlipidemia	Yes	Yes	Yes
Coronary artery disease	Yes	Yes	Yes
Prior stroke	No	No	Yes
Diabetes mellitus	No	Yes	No
Estimated glomerular filtration rate (mL/min/1.73 m^2^)	71	52	82
Procedure	Coronary angiography	Coronary angiography	Coronary angiography
Indication	Abnormal stress test and obstructive coronary disease on CCTA	NSTEMI	Chest pain and obstructive coronary disease on CCTA
Contrast type	Iohexol (Omnipaque 350)	Iohexol (Omnipaque 350)	Iohexol (Omnipaque 350)
Contrast volume (mL)	35	160	50
Access site	Right radial	Right radial	Right radial
Symptoms	Visual halos	Altered mental status, headache, agitation, aphasia, dysarthria	Bright spots in visual fields, headache
NIH Stroke Scale score	0	9	0
Imaging findings on head imaging	Normal CT and MRA	Normal CT, CTA, MRI, MRA	Punctate subacute infarct or artifact of the left cerebellar hemisphere
Clinical resolution	Yes	Yes	Yes
Duration of symptoms	60 min	72 h	35 min

CCTA = coronary CT angiography; CT = computed tomography; CTA = computed tomography angiography; MRA = magnetic resonance angiography; MRI = magnetic resonance imaging; NIH, National Institutes of Health; NSTEMI = non–ST-segment elevation myocardial infarction.

Although CIE has most frequently been reported with hyperosmolar, ionic contrast agents, our case series emphasizes that it can also occur with low-osmolar, nonionic agents such as iohexol (Omnipaque 350). These agents are still hyperosmolar relative to plasma and can disrupt the BBB, albeit less frequently. Diagnosis is primarily clinical and often a diagnosis of exclusion. Neuroimaging may be useful to confirm the diagnosis; however, it is often unrevealing. Potential imaging findings may include cerebral edema or contrast enhancement in the cortical, subarachnoid, or striatal spaces.^7^ All 3 of our patients underwent CT and magnetic resonance imaging, which were either normal or showed nonspecific findings. Given the high incidence rate and unusual clustering of events over a week period, a manufacturing, labeling, or handling error was considered. Manufacturing facilities were contacted, and an expedited internal review was performed. A temporary switch to iodixanol (Visipaque; GE HealthCare) during replacement of our Omnipaque 350 contrast inventory resulted in no further cases surrounding this time period. Other differentials including migraine with aura, transient ischemic attack, and stroke were deemed less likely in these cases given the temporal relationship to contrast administration, the presence of symptoms inconsistent with a singular vascular territory, and the negative or nonspecific neuroimaging findings. Thus, in patients with new-onset neurologic symptoms shortly after angiography—especially when symptoms are inconsistent with a distinct vascular territory and imaging is negative for other acute abnormalities, such as infarct or hemorrhage—CIE should be considered in the differential diagnosis. Management is largely supportive, there are no standardized treatment protocols, and most cases resolve spontaneously. Anecdotal use of corticosteroids, antiepileptics, or mannitol has been reported, but supportive care and neurologic monitoring are sufficient in most cases.^4^ Prompt recognition is critical to avoid unnecessary interventions.

## Funding Support and Author Disclosures

Dr Hafiz is a consultant for ReCor Medical. All other authors have reported that they have no relationships relevant to the contents of this paper to disclose.
